# Evaluation of the novel USPIO GEH121333 for MR imaging of cancer immune responses

**DOI:** 10.1002/cmmi.1526

**Published:** 2013-03-13

**Authors:** Qiaoyun Shi, Laura J Pisani, Yauk K Lee, Solomon Messing, Celina Ansari, Srabani Bhaumik, Lisa Lowery, Brian D Lee, Dan E Meyer, Heike E Daldrup-Link

**Affiliations:** a Department of Radiology, Molecular Imaging Program at Stanford, Stanford UniversityStanford, CA, 94305, USA; b Department of Radiology, Santa Clara Valley Medical Center751 South Bascom Avenue, San Jose, CA, 95128, USA; c Departments of Communication and Statistics, Stanford UniversityStanford, CA, USA; d Biomedical Imaging & Physiology Laboratory, GE Global Research CenterNiskayuna, NY, 12309, USA

**Keywords:** iron oxide nanoparticles, USPIO, ferumoxytol, GEH121333, macrophage, MR imaging, tumor imaging

## Abstract

Tumor-associated macrophages (TAM) maintain a chronic inflammation in cancers, which is associated with tumor aggressiveness and poor prognosis. The purpose of this study was to: (1) evaluate the pharmacokinetics and tolerability of the novel ultrasmall superparamagnetic iron oxide nanoparticle (USPIO) compound GEH121333; (2) assess whether GEH121333 can serve as a MR imaging biomarker for TAM; and (3) compare tumor MR enhancement profiles between GEH121333 and ferumoxytol. Blood half-lives of GEH121333 and ferumoxytol were measured by relaxometry (*n =* 4 each). Tolerance was assessed in healthy rats injected with high dose GEH121333, vehicle or saline (*n =* 4 each). Animals were monitored for 7 days regarding body weight, complete blood counts and serum chemistry, followed by histological evaluation of visceral organs. MR imaging was performed on mice harboring MMTV-PyMT-derived breast adenocarcinomas using a 7 T scanner before and up to 72 h post-injection (p.i.) of GEH121333 (*n =* 10) or ferumoxytol (*n =* 9). Tumor *R*_1_, *R*_2_* relaxation rates were compared between different experimental groups and time points, using a linear mixed effects model with a random effect for each animal. MR data were correlated with histopathology. GEH121333 showed a longer circulation half-life than ferumoxytol. Intravenous GEH121333 did not produce significant adverse effects in rats. All tumors demonstrated significant enhancement on *T*_1_, *T*_2_ and *T*_2_*-weighted images at 1, 24, 48 and 72 h p.i. GEH121333 generated stronger tumor *T*_2_* enhancement than ferumoxytol. Histological analysis verified intracellular compartmentalization of GEH121333 by TAM at 24, 48 and 72 h p.i. MR imaging with GEH121333 nanoparticles represents a novel biomarker for TAM assessment. This new USPIO MR contrast agent provides a longer blood half-life and better TAM enhancement compared with the iron supplement ferumoxytol. Copyright © 2013 John Wiley & Sons, Ltd.

Supporting information may be found in the online version of this paper

## 1. INTRODUCTION

Pre-clinical and clinical investigations have shown that pro-inflammatory leukocytes regulate the development and progression of a large variety of cancers, including lung, breast, colon, prostate, cervical, liver, ovarian, lymphoma and thyroid cancers and some pediatric solid malignancies 1–4. Tumor-associated macrophages (TAM) play a key role in this process 5. They secrete growth factors that support the proliferation of neoplastic cells, along with delivering vascular endothelial growth factor (VEGF) to activate tumor angiogenesis 6,7 and produce cytokines and extracellular proteases to support tumor invasion and metastasis 1,2,4,8–11. The degree of TAM infiltration in malignant tumors is positively correlated with disease aggressiveness and worse clinical outcomes 4,12–14. New therapeutic drugs that target TAM are currently being developed and are starting to enter the clinic 15–17. Thus, it becomes increasingly important to identify patients whose tumors are heavily infiltrated by TAM, in order to stratify these patients to TAM-depleting therapies and to monitor response to these new therapies. To serve this goal, a noninvasive and easily repeatable imaging test would be advantageous over invasive biopsy. Ultrasmall superparamagnetic iron oxide nanoparticle (USPIO) can be used for selective visualization of TAM with magnetic resonance (MR) imaging 18,19. Upon intravenous injection, USPIOs slowly extravasate across the leaky tumor vasculature and accumulate in the tumor interstitium (EPR: enhanced permeability and retention), where they are phagocytosed by TAM 20. We previously reported that significant *T*_2_-effects in tumors on delayed MR images, acquired at 24 h post-contrast, corresponded to USPIO uptake and retention by TAM as revealed by immunohistopathology 18,21. In addition, TAM depletion using specific monoclonal antibodies inhibited USPIO-induced tumor MR enhancement *in vivo* 18.

In order to translate experimental TAM imaging results to clinical practice, clinically applicable USPIO are needed. Ferumoxytol (Feraheme™) is a US Food and Drug Administration (FDA) approved iron supplement, which can be used ‘off label’ as a contrast agent for MR imaging 22–24. However, ferumoxytol has two disadvantages: first, an investigational new drug application is required to use ferumoxytol as a contrast agent, making it complicated in everyday practice. Second, ferumoxytol injection has been associated with an increased frequency of allergic reactions, most likely because of the carboxydextran coating of the drug 25. To overcome these limitations, GEH121333 has been developed as a novel, dedicated USPIO contrast agent for MR imaging. GEH121333 is composed of an Fe_3_O_4_ core and a hydroxyphosphonate–polyethylene glycol (PEG) shell (US patent 20110104072 A1). Based on the vast experience with PEG coatings in various drugs 26–28, GEH121333 nanoparticles are expected to initiate minimal adverse reactions after intravenous administration.

The purpose of this study was to (A) evaluate the pharmacokinetics and tolerability of the novel iron oxide nanoparticle compound GEH121333 in rodents, (B) assess if GEH121333 can serve as an MR imaging biomarker for TAM, and (C) compare tumor MR enhancement profiles between GEH121333 and ferumoxytol.

## 2. RESULTS

### 2.1. Pharmacokinetic and Tolerance Evaluation of GEH121333

An initial pharmacokinetic study assessed the blood half-life in rats by blood relaxometry. The relative standard error of the *T*_1_ and *T*_2_ measurements was ∼5%. The blood half-life was estimated as 2.3 and 1.8 h (based on *T*_1_ and *T*_2_ data, respectively) for ferumoxytol, and as 3.8 and 4.0 h for GEH121333.

**Figure 1:**
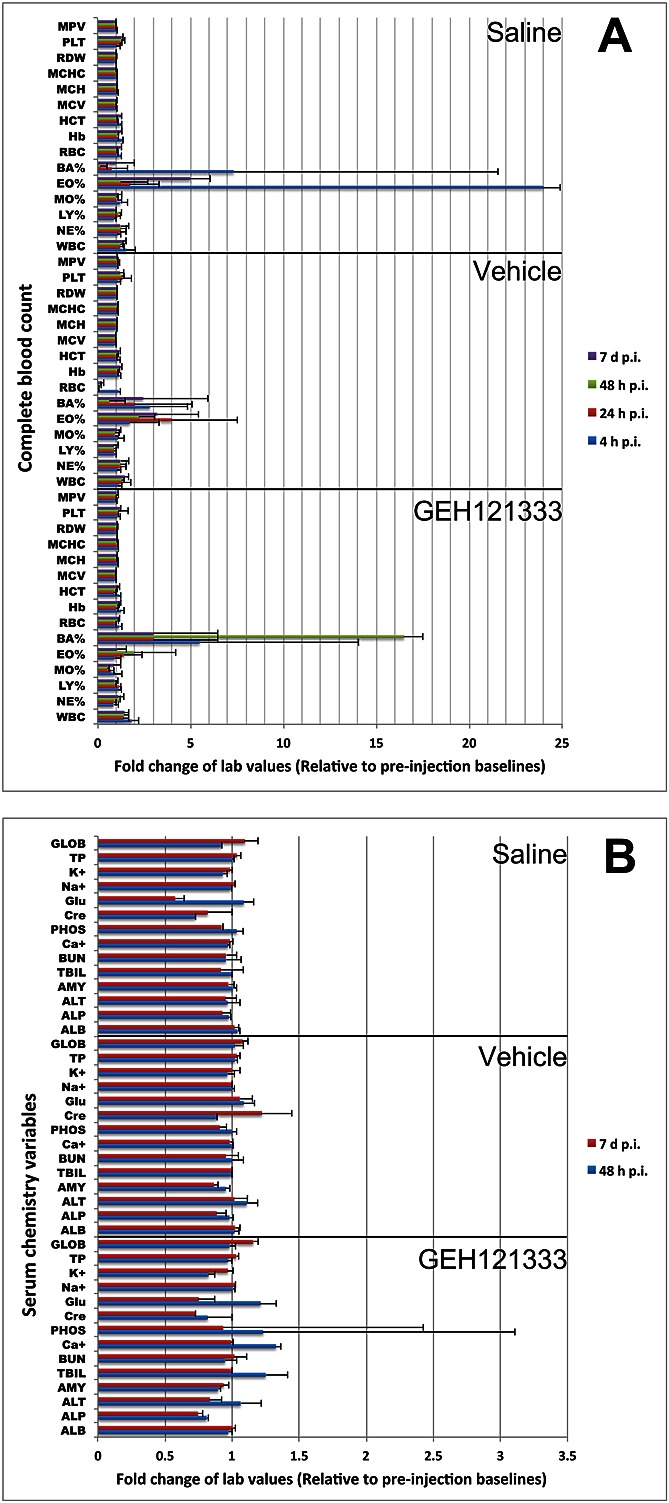
No clinically significant changes in complete blood count and serum chemistry after high dose GEH121333 injection. Intravenous injection of GEH121333 at 200 mg Fe kg^−1^ did not generate clinically significant alteration in complete blood count (A) and serum chemistry (B) profiles up to 7 days p.i. compared with laboratory values of vehicle or saline-treated animals. MPV, mean platelet volume; PLT, platelet count; RDW, red blood cell distribution width; MCHC, mean corpuscular hemoglobin concentration; MCH, mean corpuscular hemoglobin; MCV, mean corpuscular volume; HCT, hematocrit; Hb, hemoglobin; RBC, red blood cell count; BA%, percentage basophil count; EO%, percentage eosinophil count; MO%, percentage monocyte count; LY%, percentage lymphocyte count; NE%, percentage neutrophil count; WBC, white blood cell count; GLOB, globulin; TP, total protein; K^+^, potassium; Na^+^, sodium; Glu, glucose; Cre, creatinine; PHOS, phosphate; Ca^+^, calcium; BUN, blood urea nitrogen; TBIL, total bilirubin; AMY, amylase; ALT, alanine transaminase; ALP, alkaline phosphatase; ALB, albumin.

The high-dose tolerability study with GEH121333 (200 mg Fe kg^−1^) did not reveal any weight loss in GEH121333-injected rats compared with controls. During daily weight measurement study, all animals showed a slight, nonsignificant weight gain over 7 days. Complete blood count (CBC, Fig. 1A) revealed that all animals presented a transient lymphocytosis at 4 h post-injection (p.i.), with significant, 60–80% increase in neutrophils and lymphocytes compared with pre-contrast data (*p* < 0.05). There was no significant difference between GEH121333-injected animals and controls (*p* > 0.05). Serum chemistry data (Fig. 1B) did not change significantly at 48 h and 7 days after injection of contrast agent or vehicle (*p* > 0.05). We were unable to evaluate serum chemistry panels at 4 and 24 h p.i. of GEH121333 owing to colorimetric interference resulting from the discoloration of the blood by USPIO. At 7 days post-injection, no significant gross pathological abnormalities were observed in any animals treated with GEH121333, vehicle or saline. Microscopic evaluations did not reveal significant structural abnormality of reticuloendothelial system (RES) organs (liver, spleen and bone marrow), heart, lungs and kidneys in any of the animals. There was moderate Kupffer cell pigmentation in rat livers after GEH121333 administration, probably owing to contrast agent-induced discoloration. No clinically significant liver pathology was found, supported by normal liver enzymes and serum chemistry data. We also discovered mild extramedullary hematopoiesis in rat spleens, which was not significantly different between GEH121333-injected and control animals. In summary, no significant adverse effects were observed in animals treated with GEH121333 at a high dose of 200 mg Fe kg^−1^.

### 2.2. GEH121333 Produced Significant and Persistent Tumor Enhancement

**Figure 2:**
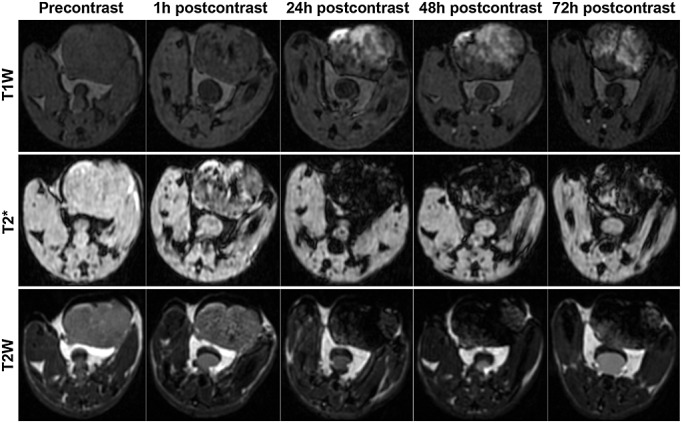
GEH121333-induced tumor *T*_1_, *T*_2_ and *T*_2_* enhancement. Representative axial *T*_1_, *T*_2_ and *T*_2_*-weighted images of mouse adenocarcinomas before and after GEH121333 injection. On early post-contrast images, GEH121333 caused mild *T*_1_, *T*_2_ and *T*_2_* enhancement. Subsequent, delayed acquisitions at 24, 48 and 72 h post-contrast administration revealed marked, inhomogeneous, and persistent tumor signal enhancement on *T*_1_ images, along with tumor signal loss on *T*_2_ and *T*_2_* images.

**Figure 3:**
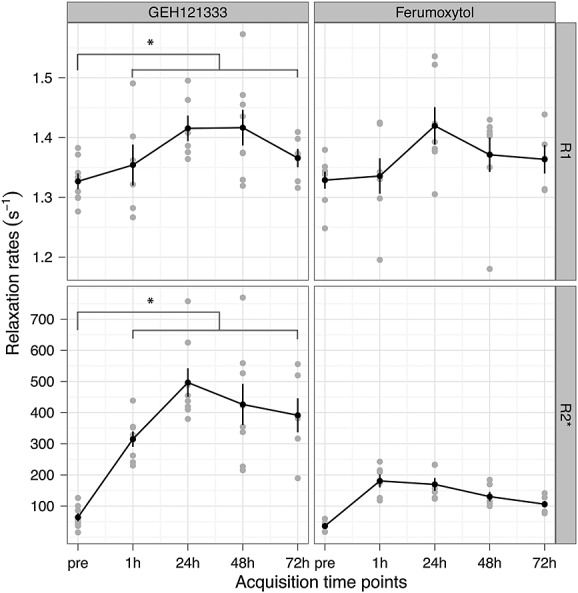
GEH121333 increased tumor *R*_1_ and *R*_2_* relaxation rates. Quantitative *R*_1_ and *R*_2_* relaxation rates of adenocarcinomas before (pre) and at different time points (1, 24, 48 and 72 h) after intravenous injection of GEH121333 and ferumoxytol are presented. GEH121333 caused a significant increase in *R*_1_ and *R*_2_* relaxation rates. Data are displayed as means and standard deviations acquired from 10 GEH121333-treated tumors and nine ferumoxytol-treated ones. The asterisk indicates statistical significance (*p* < 0.05) in post-contrast relaxation rates compared with the pre-contrast baseline.

GEH121333 nanoparticles induced a marked, inhomogeneous, and sustained tumor enhancement on *T*_1_, *T*_2_ and *T*_2_*-weighted MR images (Fig. 2). GEH121333 significantly increased *R*_1_ (*p* = 0.0291) and *R*_2_* (*p* < 0.0001) relaxation rates at each post-contrast time point (Fig. 3). Tumor *R*_1_ relaxation rates started to increase in the early post-contrast phase at 1 h, reached the maximum at 24–48 h p.i. followed by a gradual recovery that did not return to the pre-contrast baseline at 72 h p.i. Tumor *T*_2_* enhancement occurred on early post-contrast scans at 1 h, further increased up to 24 h, and persisted at 48 and 72 h p.i. No significant recovery of tumor *R*_2_* relaxation rates was found in the above post-contrast time frames. The decoupling between *R*_1_ and *R*_2_* relaxation rates at 72 h p.i. was attributed to clearance of extracellular GEH121333 (*R*_1_) and persistent intracellular compartmentalization of nanoparticles in TAM (*R*_2_*) (Fig. 5C). Histological analysis verified intracellular compartmentalization of GEH121333 by TAM.

### 2.3. Comparison Between GEH121333 and Ferumoxytol

Both GEH121333 and ferumoxytol induced *T*_1_ enhancement in tumors (Fig. 3). Post-contrast *R*_1_ relaxation rates and quantitative Δ*R*_1_ data showed no significant differences (*p* > 0.05) between GEH121333-treated tumors (Supporting Information, mean Δ*R*_1_ = 0.054, 0.217, 0.094 and 0.044 at 1, 24, 48 and 72 h p.i., respectively) and ferumoxytol-treated tumors (mean Δ*R*_1_ = 0.009, 0.094, 0.043 and 0.022 at 1, 24, 48 and 72 h p.i., respectively).

**Figure 4:**
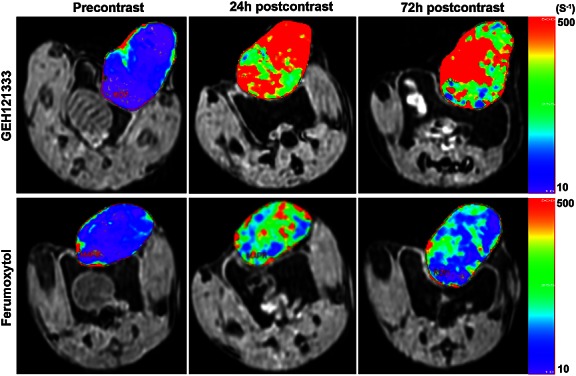
Tumor *R*_2_* maps. Representative tumor *R*_2_* maps before and at 24 and 72 h after injection of GEH121333 and ferumoxytol. The color maps correspond to *R*_2_* values indicated on the scale immediately to their right. On pre-contrast maps, tumors had relatively homogeneous, low *R*_2_* levels. At 24 and 72 h p.i., there was a widespread, inhomogeneous increase in tumor *R*_2_* relaxation rates. GEH121333 produced stronger *T*_2_* enhancement than ferumoxytol.

As shown on *R*_2_* relaxation rate maps (Fig. 4), tumors on pre-contrast scans showed relatively homogenous and low-baseline *R*_2_* values. After GEH121333 and ferumoxytol administration, there was a generalized increase in tumor *R*_2_*. GEH121333 generated a stronger *T*_2_* effect than ferumoxytol (*p* = 0.0066). Quantitative Δ*R*_2_* demonstrated a significantly stronger tumor enhancement in GEH121333-injected animals (Supporting Information, mean Δ*R*_2_* = 252.1, 433.0, 363.0 and 345.5 at 1, 24, 48 and 72 h p.i., respectively) as compared with ferumoxytol-injected ones (mean Δ*R*_2_* = 145.1, 133.8, 94.7, 70.3 at 1, 24, 48, 72 h p.i., respectively).

### 2.4. GEH121333-induced *T*_2_ and *T*_2_* Enhancement Corresponded to TAM-mediated Uptake

**Figure 5:**
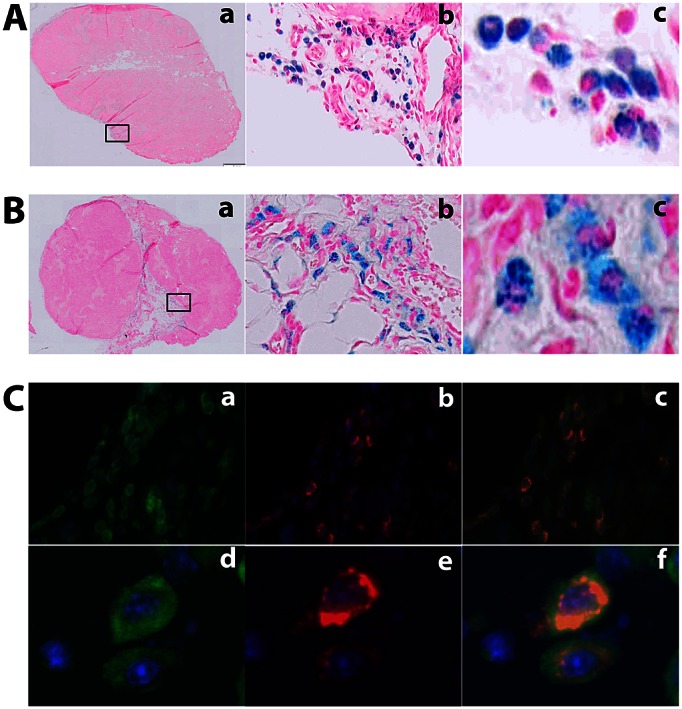
GEH121333 uptake by Tumor-associated macrophages (TAM). (A) Representative Perls stains of GEH121333-treated whole tumors at 72 h post-injection identified iron-reacting cells, mostly in the tumor periphery and around vessels in the tumor center (a, 1×), indicating intracellular iron deposition (b, 20×; c, 60×). (B) Representative Perls stains of ferumoxytol-treated whole tumors at 72 h post-injection also revealed iron-containing cells in tumor tissues (a, 1×; b, 20×; c, 60×). (C) Immunofluorescence assays using anti-PEG-Cy5 (red) and anti-F4/80-Cy3 (green) showed intracellular compartmentalization of GEH121333 nanoparticles (b, 20×; e, 60×) and the presence of TAM in the tumor tissue (a, 20×; d, 60×). Co-localization of TAM and GEH121333 (yellow/orange) confirmed cellular uptake of ultrasmall superparamagnetic iron oxide nanoparticle by TAM (c, 20×; f, 60×) 72 h after intravenous administration.

At 24, 48 and 72 h after injection of GEH121333 or ferumoxytol, Perls stain identified numerous blue-colored cells laced around the border of the tumor and in some focal areas within the tumor center, suggesting intracellular iron deposition (Fig. 5A, B). Blue-stained cell patches were often found around blood vessels. Some areas in untreated tumors also showed iron-positive cells, although far fewer than in USPIO-treated ones. Immunofluorescence assay confirmed TAM infiltration and USPIO deposition in GEH121333-injected tumors (Fig. 5C). Co-localization of GEH121333 coating (red) and TAM (green) indicated intracellular retention of GEH121333 in TAM (yellow/orange).

## 3. DISCUSSION

GEH121333 could be used as an imaging biomarker for TAM. This novel, dedicated MR contrast agent showed prolonged blood half-life and excellent tolerance in rodents and better signal enhancement compared with the iron supplement ferumoxytol.

To date, there is no FDA-approved USPIO contrast agent available for clinical MR imaging applications. Several first-generation USPIOs, such as ferumoxtran-10 (Combidex®/Sinerem®), Supravist™ and feruglose (Clariscan™), have been evaluated in phase II or III clinical trials, but discontinued owing to low efficacy, limited spectrum of applications or safety considerations 19,29–31. Second-generation USPIOs provide improved safety profiles and improved *T*_1_-contrast, thereby widening the spectrum of clinical applications. In addition, USPIOs are not associated with a risk of nephrogenic systemic fibrosis and could be used as an alternative to gadolinium-based contrast agents in patients with renal insufficiency 32. Ferumoxytol is a second-generation USPIO that has been FDA-approved as an intravenous iron supplement for anemia treatment in patients with chronic kidney disease 23. Its use in MR imaging remains off-label and investigational 33. This raises several practical issues, as detailed above, that prevent routine use of ferumoxytol for clinical MR imaging.

GEH121333 is a novel second-generation USPIO, being developed as a dedicated MR contrast agent for tumor imaging. GEH121333 shares imaging features with other USPIOs, such as initial blood pool distributuion, slow transendothelial leakage across hyperpermeable tumor microvessels (EPR), and resulting long-lasting tumor enhancement owing to nanoparticle accumulation in the tumor interstitium and TAM phagocytosis. In addition to very good tolerance in rodents, the blood half-life of GEH121333, as estimated in rats by blood relaxometry, is ∼2-fold longer than that of ferumoxytol (∼4 vs ∼2 h, respectively). This implies a slower uptake rate by macrophages in the RES, and possibly also TAM. We attribute this difference to the small size and PEGylation of GEH121333, which lead to slower RES uptake and prolonged half-life 34. To our surprise, GEH121333 produced a stronger *T*_2_* signal loss in tumors than ferumoxytol, even though the former has a lower *r*_2_ relaxivity (40 vs 89 s^−1^ mm^−1^). This may be explained by the following possible reasons: the prolonged circulation half-life and small and uniform-sized particles with monodisperse configuration of GEH121333 may lead to an increased microvascular permeability 35. The polydispersed ferumoxytol contains larger particles that locate in the tail of the particle size distribution and may show less efficient delivery 36,37. In addition, there could be a more pronounced aggregation of GEH121333 inside the macrophages.

We found GEH121333-containing TAM in patches deep inside the tumor and along vessels in the tumor periphery. This distribution is consistent with prior studies that have demonstrated attraction of TAM to hypoxic regions in the tumor center 38–40, as well as the vascularized tumor periphery, where they promote angiogenesis, and break down basement membranes and extracellular matrix, supporting vascular tumor cell invasion 38,39,41.

We recognize several limitations of our study. We used a higher (27.92 mg Fe kg^−1^) than recommended clinical dose for USPIO (5–8 mg Fe kg^−1^). This increased dose is recommended for USPIO studies in rodents, in order to compensate for the faster iron pharmacokinetics in rodents 19. Future studies have to confirm our observations in a clinical setting.

We noted colorimetric interference with serum chemistry evaluations within 24 h p.i. Of note, interference with colorimetric serum chemistry assays has also been described for gadolinium chelates, although the impact may be due less to the much faster elimination of these agents 42. Further studies are needed to clarify if and for how long interference with colorimetric assays also occurs with clinical GEH121333 applications. Clinicians would have to be educated about alternative noncolorimetric laboratory assays, which could be applied for the time period of potential interference.

As USPIOs are metabolized through endogenous iron salvage pathways, they are generally safe and have low *in vivo* toxicity 43. Likewise, our data showed that GEH121333 was very well tolerated in rodents at high doses of 200 mg Fe kg^−1^. This is consistent with safety reports of other USPIOs in the same particle size range (10–50 nm) 34,44. However, we did not evaluate the long-term *in vivo* toxicity of GEH121333. Further studies are needed to evaluate the long-term pharmacokinetics of this novel USPIO agent. Our preliminary data suggest faster metabolism of GEH121333 within the RES compared with other USPIOs (unpublished data), which could be advantageous for longitudinal evaluations.

Potential clinical applications: GEH121333 is currently being developed for clinical applications. GEH121333 nanoparticles may serve as an imaging biomarker for TAM infiltration in breast cancers and could be utilized for early therapy stratifications, to inform the development and regulatory approval of novel anti-inflammatory cancer therapies, as well as to monitor response to TAM-targeted therapies. GEH121333 nanoparticle-based TAM detection and quantification may ultimately enable a more individualized cancer treatment, leading to improved prognosis for a large variety of cancers.

## 4. EXPERIMENTAL

The study was approved by the Institutional Animal Care and Use Committees at the GE Global Research Center and Stanford University. We investigated a novel USPIO contrast agent that was developed and provided by GE Global Research (Niskayuna, NY, USA). Initial tolerance evaluations were performed by investigators from GE Global Research and the associated histopathological analysis of GEH121333-treated animals were carried out by Charles River Laboratories (Frederick, MD, USA). Imaging studies and data analyses were carried out at Stanford University. Histopathologic correlations were obtained by researchers at GE Global Research, who were blinded to the MR imaging data. Only authors who are not employees of or consultants for GE Global Research (R.M., T.D.H., S.B., S.T., S.A., G.P., M.R. and H.E.D.) had control of inclusion of any data and information submitted for publication.

### 4.1. GEH121333 Contrast Agent

GEH121333 is a new core/shell USPIO agent with an iron oxide core and a hydroxyphosphonate-PEG shell. GEH121333 nanoparticles have an average hydrodynamic diameter of 22 nm, as measured by differential light scattering with near mono-dispersity, as measured by field flow fractionation. GEH121333 nanoparticles have an *r*_1_ relaxivity of 17 s^−1^ mm^−1^ and *r*_2_ relaxivity of 40 s^−1^ mm^−1^ measured at 1.5 T and 40 °C. They undergo rapid processing from RES organs. This feature is particularly appealing when it comes to cancer treatment monitoring, which requires periodic MR imaging.

### 4.2. Pharmacokinetic Study

GEH121333 was injected through a catheter placed in the lateral tail veins of adult female Fisher rats (Charles River Laboratories, 150–200 g, *n =* 4 each USPIO compound), and was dosed at 5 mg Fe kg^−1^ body weight. The catheter was flushed with 0.9% normal saline following the injection. After 5 min, 300 µl of blood was collected into an ethylene diamine tetraacetic acid (EDTA) blood collection tube via a catheter placed in the contralateral tail vein. Sampling was repeated at 4 h post-injection. The *T*_1_ and *T*_2_ relaxation time constants were determined for the collected blood samples on a Bruker Minispec mq60 (Bruker, Billerica, MA, USA) at 60 MHz and 40 °C. A blood clearance time constant was estimated from the change in relaxation rates (*R*_1_, 2 = 1/*T*_1_, 2) over the first 4 h post-injection, after subtracting the corresponding relaxation rate for agent-free whole blood as baseline.

### 4.3. Tolerance Evaluation

Twelve 6–8 week old Lewis rats (Charles River Laboratories) were randomly divided into three separate cohorts (*n =* 4 per cohort), and received intravenous injections of either 6.7 ml kg^−1^ volume of high dose GEH121333 (200 mg Fe kg^−1^), hydroxyphosphonate-PEG, or 0.9% saline. Animals were monitored for up to 7 days after injections. Body weights were measured daily and blood was collected before as well as 4 h, 24 h, 48 h and 7 p.i. CBC, including leukocyte, erythrocyte and thrombocyte cell counts were measured with the Hemavet 750FS (Drew Scientific Inc., Waterbury, CT, USA), as per the manufacturer's instructions. Serum chemistry data (albumin, alkaline phosphatase, alanine aminotransferase, amylase, total bilirubin, blood urea nitrogen, creatinine, calcium, phosphorus, glucose, sodium, potassium, total protein and globulins) were obtained by an Abaxis VetScan VS2 with the Comprehensive Diagnostic Profile reagent rotor (Abaxis Inc., Union City, CA, USA), following the manufacturer's instructions. At 7 days post-injection, animals were euthanized by CO_2_, gross necropsy was performed and the following tissues were collected in 10% formalin for histopathological analysis: spleen, liver, kidneys, heart, lungs and bone (tibia). Tissues were sent to Charles River Laboratories for further processing, along with hematoxylin–eosin staining and evaluation by licensed veterinarian pathologists.

### 4.4. MR Imaging

Breast adenocarcinoma cells isolated from 90–95 day old transgenic MMTV-PyMT mice were implanted into the left lower mammary fat pad of 23 postpubertal, 10–12 week-old female FVB/n mice to generate breast cancer mouse models as previously described 45. When tumors reached a size of 1.5 cm, animals underwent MR imaging under isofluorane anesthesia, using a 7 T animal MR scanner (General Electric-Varian microSigna 7.0) and a dedicated single-channel transmit/receive birdcage radiofrequency coil (inner diameter of 2 cm). Animals were randomly divided into three experimental groups that received intravenous administrations of GEH121333 (*n =* 10) or ferumoxytol (*n =* 9) at 27.92 mg Fe kg^−1^ body weight (= 0.5 mmol Fe kg^−1^) or were left untreated (*n =* 4). Ferumoxytol (Feraheme®, AMAG Pharmaceuticals Inc., Cambridge, MA, USA) consisted of multidisperse nanoparticles with a Fe_3_O_4_ core and a carbohydrate coating. The nanoparticles had a mean hydrodynamic diameter of 30 nm, an *r*_1_ relaxivity of 15 s^−1^ mm^−1^ and *r*_2_ relaxivity of 89 s^−1^ mm^−1^ (1.5 T, 37 °C) 19. MR images were obtained before USPIO injection and at 1, 24, 48 and 72 h p.i., using the following pulse sequences: *T*_1_-weighted three-dimensional fast spoiled gradient recalled acquisition 6.6 ms/1.4 ms/2–30°/112 × 112 pixels (repetition time/echo time/flip angle/matrix) and fast gradient echo 6.6 ms/1.4 ms/15°/112 × 112 pixels, *T*_2_-weighted three-dimensional fast gradient echo 70 ms/1.6–20.5 ms (10 echoes with echo spacing of 2.1 ms)/20°/128 × 96pixels and 2D fast spin echo 4000 ms/42 ms/90°/128 × 128pixels (echo train length of 8). All MR acquisitions were performed with a field of view of 4.5 cm and a slice thickness of 0.6 mm.

MR data were analyzed by one independent investigator at Stanford (Q.S.) using custom research software tool (Cinetool, GE Global Research Center). *T*_1_ and *T*_2_* relaxation times of tumors were calculated based on multi-flip-angle fast spoiled gradient recalled and multi-echo fast gradient echo images, converted to relaxation rates (*R*_1_ = 1/*T*_1_, *R*_2_* = 1/*T*_2_*) and displayed as relaxometric maps. Changes in relaxation rates (Δ*R*_1_ and Δ*R*_2_*) after iron oxide administration were calculated by subtracting pre-contrast from post-contrast relaxation rates.

### 4.5. Tumor Histopathology

GEH121333 and ferumoxytol-treated animals were sacrificed for histological correlation at 24 h (*n =* 2 each), 48 h (*n =* 2 each) and 72 h (*n =* 6 GEH121333, *n =* 5 ferumoxytol) p.i. Four additional tumor-bearing mice served as untreated controls. Mammary tumors were explanted, fixed in 10% formalin, dehydrated and embedded in paraffin for histological processing. Formalin-fixed paraffin embedded whole tumor tissue sections were deparaffinized with Histochoice clearing agent (Amresco, Solon, OH, USA), rehydrated by a series of alcohol washes, and processed for iron stains and antigen retrieval. Perls iron stains of whole tumor tissue sections were performed using the Iron Stain Kit (American MasterTech, Lodi, CA, USA) following the manufacturer's instructions and counter-stained with nuclear fast red.

Immunofluorescence detection of TAM and GEH121333 in tumor tissue was performed using an antigen retrieval method. In brief, slides were washed in phosphate buffered saline (PBS) with citrate buffer for 20 min followed by 0.3% Triton X-100/EDTA for 20 min and blocked against nonspecific binding by incubating with blocking solution (10% goat serum/PBS) for 1–2 h at room temperature. A mixture of two primary antibodies containing (1) anti-PEG, rabbit-derived (Epitomics Inc., Burlingame, CA, USA) and (2) rat anti-mouse F4/80 (Serotec, Oxford, UK) was applied on the sections at a dilution of 1:100 and left overnight at 4 °C in PBS/10% goat serum. For indirect detection of bound primary antibodies, species-specific Cy3 or Cy5-conjugated secondary antibodies were used at a dilution of 1:200. The sections were further stained with 4′,6-diamidino-2-phenylindole (DAPI) to identify cell nuclei.

A pathologist who was blinded to the MR data determined whether stains were positive for iron, GEH121333, and TAM in tumor tissues, using a Zeiss Mirax Scan Microscope (Zeiss, Oberkochen, Germany) and a Zeiss fluorescence microscope with DAPI, Cy3 and Cy5 channels.

### 4.6. Statistical Analysis

Data are presented as mean ± standard deviation (SD) unless otherwise specified. Quantitative MR data of tumors were compared between different contrast agent groups and different time points before and after iron oxide administration, using a linear mixed effects model with a random effect for each animal. Results were considered statistically significant when *p* < 0.05.

## 5. CONCLUSIONS

MR imaging with GEH121333 nanoparticles represents a novel biomarker for TAM assessment. This new, well-tolerated USPIO MR contrast agent provides a longer blood half-life and better TAM enhancement compared with the iron supplement ferumoxytol.
