# Supplementary figures and images for: Evaluation of the novel USPIO GEH121333 for MR imaging of cancer immune responses
Source: Contrast Media Mol Imaging. 2013 Mar 13;8(3):281–8. doi: 10.1002/cmmi.1526 (PMC3662997; doi:10.1002/cmmi.1526)

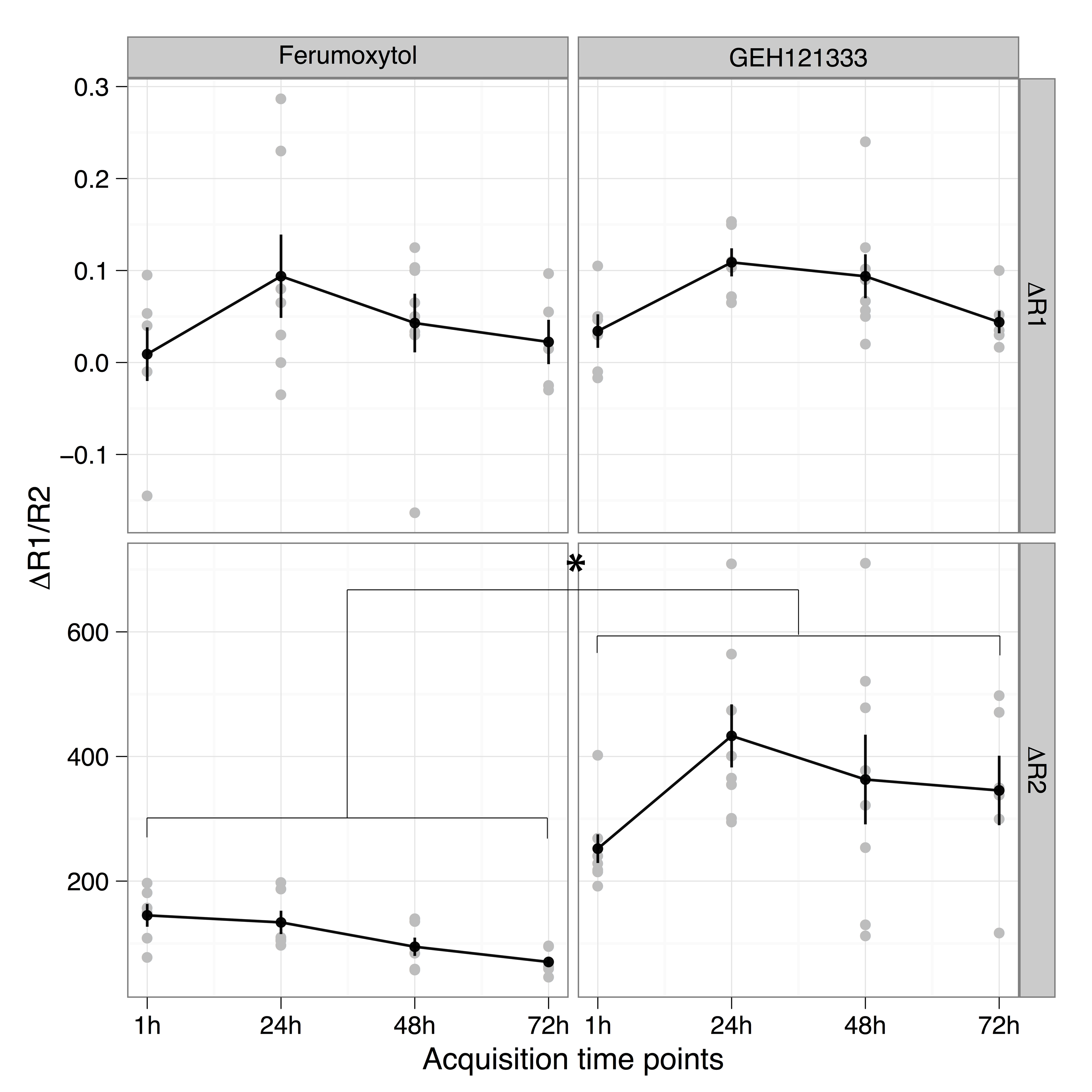

Supplement: Supplementary file 1 [file cmmi0008-0281-SD1.tiff]
